# “I can’t kill myself”: Local narratives and meanings that foster absenteeism in Nigerian primary health centres

**DOI:** 10.7189/jogh.13.04129

**Published:** 2023-10-20

**Authors:** Charles T Orjiakor, Obinna Onwujekwe, Martin McKee, Eleanor Hutchison, Prince Agwu, Dina Balabanova

**Affiliations:** 1 Department of Psychology, University of Nigeria, Nsukka, Nigeria; 2 Health Policy Research Group, College of Medicine, University of Nigeria, Nsukka, Nigeria; 3 Department of Health Administration and Management, University of Nigeria, Nsukka, Nigeria; 4 Department of Health Services Research and Policy, London School of Hygiene and Tropical Medicine Faculty of Public Health and Policy, London, England, UK; 5 Department of Global Health and Development, Faculty of Public Health and Policy, London School of Hygiene and Tropical Medicine, London, England, UK; 6 Department of Social Work University of Nigeria, Nsukka, Nigeria

## Abstract

**Background:**

Absenteeism in the health sector is increasingly seen as a form of harmful rule-breaking, with health workers receiving a salary although they are not present to provide care. It is a barrier to achieving universal health coverage yet remains widespread in primary health centres (PHCs) in Nigeria and many other low-resource settings. Traditional approaches to combatting absenteeism have relied on anti-corruption measures such as promoting accountability and transparency. However, more needs to be understood about the social and cultural realities, including perceptions and norms enabling or constraining the application of such measures in Nigeria and in similar contexts.

**Methods:**

We conducted 34 in-depth interviews (IDIs) with frontline health workers and their managers/supervisors and two focus group discussions (FDGs) with service users (n = 22) in Enugu State, South Eastern Nigeria. We discussed their experiences and views about absenteeism, allowing the respondents’ framings to emerge. We adopted a mixed approach of narrative analysis and phenomenology to examine respondents’ narratives – identifying the concepts and social constructs within the narratives that manifested through the language used.

**Results:**

Stakeholders acknowledged the problem of absenteeism but had differing perspectives on its dynamics. Health workers distinguished two forms of absenteeism: one as a mundane, everyday response to the poorly funded health system; and the other, brazen and often politically enabled absenteeism, where health workers whom powerful politicians protect are absent without facing consequences. There is a general feeling of powerlessness among both health service providers and service users confronted by politically backed absentees as the power dynamics in the health sector resonate with experiences in other spheres of life in Nigeria. Health workers rationalised mundane, technical absenteeism, adjusted to it and felt it should be accommodated in the health system. Service users are often unsure about who is absent and why, but when they notice absenteeism, they often ascribe it to wider system malpractices that characterise public services.

**Conclusion:**

Interventions to tackle absenteeism and other forms of health sector corruption should be sensitive to socio-cultural and political contexts that shape everyday lives in specific contexts. Challenging narratives/beliefs that normalise absenteeism should be part of reform plans.

Rule-breaking in the health sector takes many forms, from absenteeism to informal payments, supply chain and employment irregularities. These different types of behaviour constitute corruption and threaten access to lifesaving services, with consequences for access to health care and resulting outcomes [1]. Although corruption is a global problem [2,3], its impact is often disproportionately felt in low and middle income countries, where there are fewer resources, affecting poor and vulnerable groups disproportionately [4], although corruption is found even in highly-regulated health systems with effective systems of oversight [5,6]. The coronavirus disease 2019 (COVID-19) pandemic revealed many such examples [7,8].

Corruption has traditionally been understood as a technical issue to be managed using good governance policies and the rule of law, strictly enforced regulations, and robust systems of accountability in the public sector [9-11]. However, it is increasingly recognised that these approaches have not been successful [5,12], leading to a new generation of studies asking how contextual factors, including the political economy and social networks, shape rule breaking and rent-seeking.

Jackson and Köbis suggest that lack of understanding of contextual factors such as local social norms could be a reason as to why many initiatives and strategies that promote accountability and integrity do not lead to tangible improvements [13]. Poorly targeted anti-corruption information and messaging campaigns also fail to garner support, and in some cases, make the public more despondent about tackling rule-breaking and rent-seeking [14]. Anti-corruption strategies that are sensitive to contextual peculiarities are thought to be more likely to have an impact [12,13]. Strategies that take into account the socially-constructed nature of corruption, and the local interpretations and reactions to rule-breaking behaviours are more likely to be successful [15,16].

One way to take account of the socially constructed nature of corruption is to investigate the language and stories used to describe it [17]. Narrative analysis begins with recognising that people organise and make sense of their lives through functional and purposeful stories [18]. The analysis of the structure and content of life histories, diaries, and letters as narratives has been particularly useful in making sense of how power operates within society and how gendered relationships impact individuals and groups [19]. Narrative analysis provides additional depth to the growing literature on social norms and corruption, which is similarly interested in everyday assumptions and concepts that unconsciously structure patterns of behaviour.

In this paper, we examine how staff absenteeism in the health sector is understood and imbued with meaning by health service providers and service users in Nigeria. We applied qualitative analytical methods to a series of in-depth interviews (IDIs) and focus group discussions (FGDs) and seek to trace the constructs behind them and how they may motivate (in)action on the part of these two groups. We conclude by considering the implications of these narratives for tackling absenteeism in the Nigerian health system.

## METHODS

### Conceptual framework

Since we were interested in understanding health worker absenteeism in relation to the social norms that underpin their attitudes, beliefs, norms and meanings shaping their decisions to be absent, and the experience of service users and other actors involved in the health system, we sought to explore the dominant narratives among these groups. We adopted two related approaches that focus on narratives: narrative constructionism [20] and Darren Langdridge’s critical narrative analysis (CNA) that draws from hermeneutic phenomenology [21,22].

Narrative constructionism considers the social and cultural realities that individuals deploy in describing and communicating their personal experiences [23]. In the current study we specifically focused on and used narrative analysis. Narrative analysis entails the use of life stories derived from diverse sources including interviews, observations, and discussions about specific people, places, or events [24-26] in harvesting knowledge about the nature of a phenomenon. We utilised data from interviews and FGDs for the current study.

CNA is a less popular phenomenological methodology that focuses on narratives (life stories) and the description and interrogation of social realities [21]. CNA was adopted as a complimentary approach because of its fittingness in deconstructing meaning within social contexts, as well as being appropriate where power and politics are expected to be at play. It has proven useful in interpreting local parlances used within local contexts.

### Study area, sampling and sampling size

Using purposive sampling, we identified 40 primary health centres across four local government areas in Enugu State, Southeast Nigeria. Participants were 27 frontline health workers working in 10 primary health centres (which included two doctors, ten nurses/midwives and 15 community health workers); four heads of health departments (HODs) in the four local government areas; three health supervisory councilors; and 22 service users which included seven members of health facility committees. Thirty-four IDIs and two FGDs were conducted. Participants were aged between 24 and 68 years.

It is important to note that the management and running of primary health centres in Nigeria is the responsibility of local government (LG), which is responsible for drafting and implementing a range of policies to coordinate activities at the grassroots level. Thus, the LG oversee activities that include marketplace administration, local road maintenance and primary health centre management, including the payment of wages. Each LG authority has departments corresponding to the Ministries at the State and Federal levels, of which the health department is one. Each department is headed by a HOD and a supervisory councillor. While HODs are career staff, the supervisory councillor is a political appointee, selected by the local government chairperson. Primary health centres (PHCs) facilities have facility committees comprising representatives of the local community working with the facility’s leadership.

### Data collection instruments and data collection

Ethical approval was obtained from the University of Nigeria Teaching Hospital Ituku Ozalla (Approval no: NHREC/05/01/2008B-FWA00002458-IRB00002323). The development of pilot interview and focus group guides that focused on the occurrence, nature, and drivers of absenteeism in PHCs was informed by a systematic review on health sector corruption in Nigeria [27] and a consensus-building exercise with frontline health workers and top health policymakers on the nature of the problem and possible solutions in Nigeria [28]. In line with the requirements of narrative analysis, questions in the topic guides were open-ended with probes and prompts used to follow up stories of interest. Participants were urged to tell a story of real-life experiences of absenteeism. Follow-up questions tried to elaborate the story by querying who was involved, when, and why the person could act in such manner. We conducted a pilot study involving 24 frontline health workers (six health managers, six doctors, six community health extension workers) and six service users in different locations away from those we selected for the main study. Findings from the pilot were analysed during several research meetings and the final guides were developed. The scope and depth of questions were also reviewed by three health system and political economy research experts. In line with narrative analysis, items in the guide were refined to be more robust, to capture the nuances and dynamics of absenteeism in PHCs.

The director of the Enugu State Primary Health Care Development Agency (SPHCDA) permitted researchers to access health facilities and contact staff to enrol them in the study. Four local government areas were selected, representing facilities in urban and rural areas. Twenty health centres, five in each local government area were randomly selected and visited, and health workers in the health centres were recruited for the study. Consenting health workers across the facilities visited were interviewed. Through the health facility committees of the facilities drawn for the study, 15 community members were invited to participate in FGDs. Participants received 500 naira (about 1 US dollar) to cover their transportation expenses. Participants interviewed during the coronavirus disease 2019 (COVID-19) pandemic also received face masks and hand sanitisers. The duration of interviews varied, lasting between 13 minutes and one hour. FGDs lasted for around one-and-half hours. Data collection was conducted between January 2018 and August 2020. The lead author returned to the field to follow up with specific respondents, gain clarification, or elicit more details about stories.

### Data analysis

Interview and FGDs transcripts were transcribed verbatim and prepared for coding. Interviews conducted in the local language Igbo were translated and transcribed by research team members from the local setting. The coding process was inductive to allow for germane and little theory influence in deconstructing the meanings emerging from the narratives of the respondents. Coded transcripts were shared across the research team members who debated in research meetings until a consensus was reached on the themes, descriptions, and interpretation of narratives.

## RESULTS

In the following sections we present the dominant narratives created by the respondents. Although health worker absenteeism was found to be a common manifestation of nationwide multisectoral dysfunction, there are unique local understandings and rationalisations – particularly on the reasons why health workers are absent. We identified common phrases or parlances used to describe and discuss absenteeism and other forms of corruption and sieved out what they meant to respondents. Narratives illustrated the underlying drivers, including the illicit behaviours of actors, ill-behaved health workers, power hierarchies (both political and communal), impunity, and system inefficiencies. However, participants’ narratives still recognise that absenteeism is considered poor and counterproductive behaviour, and these appear to be associated with negative feelings towards absent staff.

### Absenteeism is ubiquitous

Generally, respondents accept the narrative that depicts absenteeism as a manifestation of a systemic breakdown affecting all sectors across the country, where harmful rule-breaking and malpractice are shielded and tolerated. This inability to guarantee law and order, rooted in political protection, was acknowledged as weakening enforcement mechanisms. The protection of defaulters becomes normalised. This is reflected in the narrative of this health manager:

*So…you know local government has a problem and the problem is not only in health, there is a problem in every department in the local government. These things that are happening everywhere… Somebody will be with you, will be committing atrocities and …, you can’t do anything. The problem …is everywhere, it is still in the health sector, it is still in every other sector within the local government. But especially in the health sector.* (Male, Officer in charge, (OIC) 45, IDI)

Respondents described absenteeism in many of the conversations we had as a common, almost everyday occurrence. One service user described how: *“two women in labour came from a far distance just to find no one at the health centre”* (Female, 31 years old, FGD with women). A health supervisor also reported complaints from a community she covered*: “the community complained about the nurses who are often absent during night shifts…”* (IDI, supervisory councillor). Some described it as somewhat predictable – a malady characterising many public offices in the country. Few people express any sense of ownership of public facilities and many staff adopt a lax approach to absenteeism. However, this does not mean that the absenteeism sits well with service users and even coworkers as shown in the subsequent narratives.

### Absenteeism can be hidden

It was surprising that communities suffering from absenteeism may only sometimes be aware of its extent unless it becomes extreme, for example, when the health centre is shut down. Communities and their representatives may be unaware of the intended staffing levels in health facilities, or the number of staff required to be on duty on any given day. Communities, therefore, begin to worry only when the health centre grinds to a complete stop,

*...The community won’t know who is on duty and who is not... they may not have time and may not know what is happening in the health facility,... once they see someone that will render services to them they will think that everything is fine.... What you are asking may take place in cases where the health centre is deserted..., and...it is common. That is when the community will ask, “what are you people trying to do? are you trying to lock down our health centre?”* (Male, OIC, 45 years old, IDI)

Only health workers in health centres know the official schedule. This enables health workers to structure shift patterns in ways that are favourable to them but may also compromise quality and timeliness of care. It is important to note this as service users and sometimes supervisors only see absenteeism when they do not see anyone in the health centre and may not understand its full scale. What appears to matter to the service users is that they see someone to attend to them when they arrive at the facility. It may mean little to them that only a few health workers were ready to render services or that services were delayed compared to a total absence of workers in a health facility. The narrative of hidden absenteeism occurs in settings where the actors assigned to monitor health facilities are poorly engaged – for example, not actively seeking to find out who should be in work at any point in time. Health workers then capitalise on this low interest to conceal and normalise absenteeism.

### Negative emotional sentiments about absenteeism

Although absenteeism, whether obvious or hidden, is described as common and cuts across sectors of society, it is important to note that narrators often described it with negative and disapproving sentiments. They often express their disappointment, dissatisfaction, and annoyance about experiencing it. One service user described her reaction to finding no health workers in the health centre: *“I feel bad and if I did not see any worker, I will go home very angry...”* (female FGD participant, 38 years old- service user).

Sometimes the situation was described with a sense of hopelessness. A health worker talked about her frustration with the absence of colleagues and how the situation seemed hopeless: “…*If you come and scream up and down, for him to come to work, it doesn’t concern him… The person will take your admonitions as nothing*” (IDI with Female OIC, 50 years old).

These narratives portray a widely shared understanding that absenteeism is not palatable and that both health workers and service users were unhappy and dissatisfied with such practices.

### Sheer stubbornness and arrogance

Some workers were described as having a poor attitude to work, which manifests in neglecting their duty. Non-compliant health workers would typically engage in absenteeism and refuse to cooperate with health managers. One OIC described that *“…some people (health workers) are arrogant in nature*”. Sometimes workers come up with nonchalant mannerisms and act in daring ways. Another health facility manager gave a more elaborate narrative that describes the extent to which some health workers can be callous and unrepentant about being absent from work:

*Like in my health centre, if someone is usually absent and you query the person and tell him or her you will send the query to the HOD, they will ask you if you are the HOD who employed them. Some of them are school teachers, others are in private businesses, yet still holding (this) the job. There is another that works at XXXX (a popular eye clinic), another at XXXX (a health facility run by a faith-based group)… one produces soap and supplies to different places around. She will pass through the health centre and hail me “Boss!” And I will just hail her back “my sister my sister”!* (Female OIC, 52)

The sheer arrogance and disregard of the health worker who goes about her private business while being absent from their duty post while breaking the rules points to obvious distortions in power relations between the daring health worker and the powerless health facility manager. This power dynamic is further unravelled in subsequent narratives.

### Absenteeism, politics and power

There are absentee health workers who are protected by politically powerful figures and/or groups, and this means that health managers and sometimes community representatives are powerless to act against them. The narrative of powerlessness is dominant among those expected to provide oversight. Powerlessness also surfaces in discussions with coworkers and service users. Health managers are unable to exercise control over subordinates who are connected to “*people at the top”* (IDI with OIC). Because health managers often feel and act powerless when a worker has political backing, anybody with the backing of a sufficiently powerful person(s) can be absent, confident that they will not be sanctioned. A facility committee chairman observed that: *“…such people are very difficult to sanction because of the people they have at the top*” (IDI with health facility committee chairman). The powerlessness felt by health workers and facility managers leads to a perception that it is pointless to press absentee health workers who benefit from patronage relationships. One health facility manager narrated her experience when she tried to follow up an absentee staff with sanctions: “*like someone we issued query, she had a godfather, the query was not answered and before you know it. A call from above said leave that person…and there is nothing you can do about it*” (female, OIC, 45).

In Nigeria, the roles that political networks and connections play are widely discussed in many spheres of life including work settings [28]. Political affiliations influence who is employed, who can access highly sought resources, such as prized training opportunities within the workplace, and who can be sanctioned when rules are flouted. Crucially, the narratives used by respondents regarding this problem indicate that it is not peculiar to the health system, but part of the fabric of Nigerian society. People with power, especially political, are considered untouchable. They were commonly referred to in many narratives as “… *somebody at the top*” (male, health facility committee (HFC), IDI). Those “people at the top” are looked at with fear that they can make or mar people who are insubordinate to their bidding. A facility health manager described how commonplace and cross-sectoral the political influence is:

*So, … the problem is not only in health, there is a problem in every department .... That is protection…you know, people will do something that… require to be punished…they will be shielded from the punishment….* (OIC, male, 52)

The absences of health workers with political connections are typically brazen and long-standing. The political protection enjoyed by certain health workers helps to normalise their absences as their superiors and colleagues feel powerless to address them. In fact, an enforcer may suffer backlash from powerful people involved. This is illustrated by a health manager who points to why their colleagues in management positions may be unwilling to enforce sanctions because “*they may be afraid that the person in question has some body that is beating drum for him or her*” (male OIC, 48, IDI). This phrase is derived from a local proverb that a bird dancing in the centre of a path must have someone beating the drums in a nearby bush. The effrontery of a dancing bird in the middle of a path connotes an unprecedented boldness to act in otherwise dangerous ways (here, leaving their posts without permission) and the hidden drummer refers to the hidden backer. Thus, it suggests that those who enjoy protection from sanctions when they absent from work have the support of unseen, but powerful actors.

### Political protection-an open secret

Powerful people who protect absentees are often not directly seen or known; instead they were described as acting ‘in the shadows’ where they pull political strings which can embolden deviant workers. Deeper probing was often required to find out who they are. Interestingly they are a diverse group, but politics and power are common underlying factors in their identities. A facility manager commented that: “*they are all politicians*…” (HFC, IDI). Another facility manager described them to be “…*any politician or any senior civil servant in the local government*” (male OIC, IDI). A vaccine officer gave an example: “*they have people even in the House of Assembly (legislative body of the state government), those people cover them*” (female vaccine officer, 48, IDI). Sometimes the powerful person may be a relative of the deviant health worker as described by a health officer: “*something like that, a husband, and that husband happened to be in a political position*” (female HOD immunisation). Often, they have played a role in recruitment and employment of the transgressing health workers and have continued to protect them: “*maybe they have godfathers or somebody that helped them secure employment. You know that the person will not allow his own to suffer*” (female OIC, IDI, 52 years old).

Political protectors do not offer their protection openly. Their interference is often indirect, for example through calls to persons in higher authority who can directly influence the actions of line managers. These high-level protectors often do not want to be seen to endorse wrongdoings because they understand that it could have a negative impact on their image if this goes public. Interestingly, key players often seem to know who has protection but may not exactly know who is offering it.

At other times narratives convey how workers seek protection from powerful people. One health manager described some workers as having “*long legs… they can penetrate where you cannot imagine*” (female HOD, Immunisation, IDI, 48 years old). This mastery in permeating high places, influencing and befriending persons in authority often describes how health workers get the backing of powerful people.

### Feared losses from addressing absenteeism

Powerful people and forces fostering absenteeism extend beyond politicians, and support for health workers can sometimes come from unusual places. In a setting where recruitment of health workers is rare and may happen only once in ten years, communities and other significant actors may sometimes act to protect deviant health workers from sanctions. They recognise that the worker is a member of the community and sanctioning them will ultimately create problems for members of their family or other people from the community who depend on them for essential services. Take this narrative from a health manager:

*… There will be divisions within the community, (between those) who want to favour this person and who want to kill (penalise) this person. Some people will want to kill her ehn… some people will say no, no leave her ehn how much is she even paid? He is our child we will not treat him so. He is a member of our church we will not do so to him. He is a member of our group; we will not treat it like this. He is in our forum, you can’t do it that way. That’s the problem, you see?* (male OIC, 48, IDI)

It is interesting that enforcing sanctions as a form of punishment was described using words such as “kill”. Sanctions are seen as depriving a health worker of their livelihood and when it involves a member of the community who has defaulted in their duty, community representatives relent from implementing sanctions. One can also perceive, in the quote above, that a deviant health worker is referred to as “a child” or “our child”. This demonstrates that when health workers are members of a community by birth or have other social bonds within it, s/he is recognised as “one of us” and sanctions may be withheld, offence minimised or inaction rationalised. This ambivalence toward absentee health workers may mean that transgressions are not punished, which may encourage further rule-breaking given the mixed message in the dominant narratives and the local support.

### The fight to counter the powerful

Actors’ narratives also touched on the power and ability to push back at powerful persons, especially when one is in the position to, and has the courage and wherewithal. One means is “query writing”. In the Nigerian public service context, queries are official letters by a senior official to a subordinate, regarding a specific perceived misconduct; asking for explanations and why further disciplinary actions should not be taken against the subordinate. Queries are documented in employee folders and could be used as an indicator of conduct and performance for future decisions; for this reason, no one wants to receive a query. A manager in a health centre revealed that query writing worked to compel health workers to behave adequately if accompanied by sufficient courage and knowledge:

*…Yes it (query writing) works, when the person that is involved has the liver to write the query. But em, I know quite well that people are protecting people, but you see, when you know what you are doing, you can write. Unless when you’re afraid of writing or maybe you don’t know what to write.* (male OIC, 48, IDI)

“Having the liver” in the local Nigerian parlance implies “having the guts” or being bold enough to take on a challenge head on. The exact opposite of being lily-livered in these situations. To confront powerful people, one needs the liver, the courage to act – and there is a clear understanding that acting against rule breakers can potentially have serious adverse consequences. Sometimes strong-willed actors willing to deter absenteeism may involve equal or higher-ranking persons that can neutralise the political backing of the absent health worker.

In another case, a female health facility manager stood her ground against a local government administrative boss who wanted to exonerate chronically absent staff from impending sanction. The narrative elicits the dangers for the respondent who seeks to counter patronage relationships.

*One of the very last verifications – two of these staff came in company of the HOD immunisation …to pick the register from the table of the officers doing the verification for us. What will you do to them? Seeing they were led by the HOD, sent by the vice chairman? You will only think there is an official need for it. But I noticed it was a shoddy thing (they want to put their names when they were absent) and I jumped at them and collected the register from them…He (the vice-chairman) collected the register from me and asked me what I want to be done? I told him to do whatever was best. He started calling me idiot, bush animal, you alone will go to heaven and others will not. I told him that they need to be adopting good leadership qualities to motivate staff to do the right thing. He started asking me do I know whom I am addressing in that way?... They know for over six months they have been away from work…. The vice chairman tore off those pages of the register where their absence were recorded and he wrote a fresh attendance list with their names all added.* (Female OIC, 55)

In this explicit confrontation – which is rare as conflict is often channelled through indirect means, we see how the high-ranking official (the vice chairman) tries to blackmail the health manager (using abusive language) into accepting that she is being insensitive, and in the wrong. Insisting that the names of absentees should not be in the register was interpreted by the official as trying to go “to haven alone”, or rather, preventing others from going to haven i.e. receiving pay or avoiding punishment. In this expression he suggests that the health manager is being an impediment to the “goodwill/wellbeing” of others. Workers who are absent often use emotional blackmail to compel health managers to not be hard, as they are not the ones whose money is personally spent on paying absent staff. Another underlying meaning that can be deduced from the encounter presented above is that pay/wages, especially in the public setting, is not often associated with work/in-put, but as a benefit or handout from the government coffers. This is also a common perspective in Nigeria.

Another point from the confrontation depicted in the above quote is the rhetorical question posed by the official – “do you know who I am?”. This is language that officials and powerful persons commonly utilise in pressurising those lower down in the hierarchy into subordination. It is often a veiled threat rather than a question. Thus, the official was trying to threaten the health manager into submission to enforce his wishes.

### Reciprocal relationships

Absenteeism was also seen to arise from transactional behaviours between health workers and health managers. A distinct narrative emerged: absenteeism was part of a complex web of mutual favours and interrelated corrupt practices. Health workers may offer bribes to higher-ranking health workers/managers to cover their absenteeism. One OIC narrated how she was accused of taking a bribe because she did not report a chronically absent health worker.

*One of my workers stopped coming to work… So now when our supervisors came for series of supervision… they called me and said maybe I am taking bribe from the staff if not, why can’t I tackle and report the person… why is it that this person has not been coming to work this and that… So, when I started asking questions, I realised that there are some who are absent because they bribe people to cover them up?* (Female OIC, 49, IDI)

The narrative reveals the suspicion of supervising officials, given the deviant means through which absenteeism is perpetrated by health workers, and their attempt to uncover it. This interaction between supervisors and facility managers raises more awareness on the previously unknown means by which absenteeism is perpetrated – such as bribery. Health workers also describe the challenges they face with stubborn colleagues who hold down many jobs and which hampers the work of the health centres. Another OIC describes an incident where a HOD struggled to defend her integrity as she was reported to have collected bribe to cover health worker’s unauthorised absence:

*…That staff was heard telling others that, “this person reported me to the HOD that I’m not going to work and I just used money and settled my HOD and that was the end”… So it wasn’t easy because the HOD was trying to save herself she said “such a thing never happened” … But we know such things do happen.* (Female OIC, 47, IDI)

These reports evidence the reciprocal pattern of behaviours that supports absenteeism across hierarchies. The perpetrators are known and at the same time hidden.

### When absenteeism solves problems

In contrast to the anger and fear that developed around absentee health workers protected by powerful political networks, some “acceptable” circumstances in which absenteeism may be tolerated were discerned in the narratives of service users and health workers. Often these reasons for absenteeism are communicated in ways that are admissible or permissible given local realities. Reasons slightly differed between health workers and service users as described below.

#### Poor pay and inadequate working conditions validate absenteeism

Health workers’ narratives around absenteeism are sometimes framed that it occurs in response to external constraints. It could be driven by low wages or other health system deficiencies that can work against them, causing them to be inevitably absent from work. Health workers then understand that they must find ways around these system problems. One unintended outcome is that they are often absent from work on occasion. This is depicted in the quote below where a manager recognises that workers need to ‘make ends meet’:

*...The money they are getting is not commensurate to the work they do. Sometimes, they try to fix themselves in other business(es) so that they can make ends meet. So they will not be able to give full attention to work and sometimes they are busy trying to fix themselves instead of being in the health centre* (Male, 42, HOD, IDI).

An OIC narrates how her workers urged her to organise the schedule in the health facility to enable them to earn extra income from other engagements outside of their official duties. Workers often cite the poor wages and further justify their stance with other facilities also adopting pragmatic approaches to work scheduling:

*Sometimes, you start hearing stories, see how others do their own, ehh you want us to be coming every day to work. This and that, that thing they pay you, is it enough to cater for your needs? Why can’t you look for another thing and be doing to augment your salary?...* (Female, 44, OIC, IDI)

Public sector wages are often considered low, hence the derogatory description of wages as “that thing”. Thus, health workers seek to adjust the shift patterns to give them time to do other jobs to augment their pay. In addition to acknowledging the problem of low earnings, and the need to augment salaries, a clear theme from the above quote is that health facilities can organise locally, irrespective of formal governance mechanisms and political forces that impinge on them. One subtle but common parlance depicted in the above quote is “how others do their own”. The health manager (OIC) in the above narratives captures the pressure from her subordinates who are urging her to consider how other health centres have remodelled their shift patterns. Often health workers adjust shifts so that fewer than the required health workers will be at work during a particular shift. The ‘freed’ health workers can then go ahead to take on other jobs or activities that can help them support themselves. Thus, absenteeism is normalised and seen as secondary to the imperative to safeguard family livelihoods, for example through having a second job.

Another health worker recognised that poor transportation services may make workers walk huge distances, leaving them exhausted from the journey: *“they come to work and start saying my waist, I trekked and my legs are aching me, because of that they lie down in the facility…you know… I can’t kill myself…”* (female OIC, 51, IDI).

Commuting to health facilities can be very challenging particularly in rural and hard to reach places. As transportation to these areas is difficult, health workers may take time to recuperate. Another striking parlance common amongst Nigerians is demonstrated in the above quote – “*I can’t kill* myself” – commonly used to denote that one should not endure extreme suffering in carrying out their own duties. This narrative also exemplified presenteeism, where workers are physically present in the workplace to avoid backlash but do not engage in productive work such as attending patients.

In some cases, absenteeism is seen as a logical response to systemic inefficiencies and poor infrastructure but at the same time there is awareness that it may lead to low utilisation for health centres, causing health centres to adjust their schedules in response.

*…We have sensitised everyone to make sure they report to work regularly because we normally have shift arrangements. Like Mondays, everyone reports to work …and other days we can say two will be in, others will rest… We don’t even have patients because of the poor road network and other things of which we have severally reported to the government. Just like you can see for yourselves (gestures to the quiet health centre). Assuming it rained, like yesterday or even two days ago, you people would have found it difficult reaching out to us here but you are lucky you came with a big car. To some after visiting, we have to push their cars as a result of how inaccessible the roads are.* (Female health worker, 26)

## DISCUSSION

Despite widespread reports about absenteeism in the Nigerian health sector and in many other low and middle income countries, the problem seems intractable. Even biometric attendance monitoring systems have not helped to reduce health worker absenteeism [27], as local conditions made the operation of these devices impractical. The ubiquitous nature of absenteeism and the lack of evidence of what works to reduce it has normalised absenteeism of health workers in many settings [29]. Our narrative analysis approach focused on the perception of absenteeism by those involved in with or affected by it. We explored how health workers, health facility managers, supervisors, and service users create meaning around absenteeism and how they respond to different forms of absence. This revealed the social norms and local perspectives that intersect with governance measures, which may help to explain why absenteeism persists.

We found that first, absenteeism was not considered peculiar to the health sector. A general breakdown of law and order in the local government administration and the wider Nigerian society was identified as underpinning absenteeism and indeed other vices. General deficiencies in national accountability frameworks and processes influence its occurrence within the health sector. The collective action theory describes how people break rules simply because others are doing it [30,31]. Acknowledging widely held beliefs that everyone is culpable – “everyone does it” – and challenging the normalisation of rent-seeking behaviour such as absenteeism, should be a critical component of strategies addressing it. Showcasing examples of rule-following health workers and incentivising them could be a good way to challenge widely held counterproductive beliefs.

We see from the narratives around absenteeism that its meaning and how it is rationalised differ. Socially ingrained conceptions of the power wielded by the political class or other people in authority, as well as other social nuances, defined how absenteeism is seen and dealt with, both at the facility level and within local communities. Health workers who can avoid sanctions for absenteeism disadvantage less powerful colleagues, frontline health managers, and service users. These powers draw from societal definitions of what power is, who wields it and what are the consequences of opposing it. A recent study highlighted the political class’s role in enshrining absenteeism in the health sector [32]. The powerful, often the political class, are seen to be “on top” of societal affairs, controlling what happens at the frontline, and have the capacity to protect or punish as they please. This perspective is generic in Nigerian society, across most sectors, and influences how rules and regulations are enforced. Powerful members of the community/society (often from the political class) bear down on health managers to ignore or tolerate absenteeism perpetrated by their ‘candidates’ who are health workers. Often, health managers decline to enforce sanctions, fearing a backlash from powerful politicians. However, the definition of who is powerful is relative. We found that, apart from the political class, health managerial hierarchies offered protection to absentee health workers when they can benefit from it. The local understanding, ingrained within stakeholders’ norms and expectations, is that anyone that has a powerful backer can be absent without sanctions. Health workers who do not have the backing of powerful people are left to carry heavy workloads to sustain services in health centres. Navigating around the political class (and hierarchies) is critical to combating absenteeism, but clearly solutions need to be designed at the community level taking account of the local political economy.

Although the conception and use of (political) power is predominantly identified as a factor in health worker absenteeism, other nuances help normalise it. The scarcity of jobs and resources means that communities may also rally to protect health workers who share family or social bonds. Health centres' sanctions for absenteeism (and other wrongdoings) often target (or impact upon) the pay of erring health workers. Communities and coworkers realise that allowing sanctions to fall on one of their own means that some members of the communities who are dependent on that health worker will lack means to livelihood. Communities then rally to protect health workers who have transgressed. Rooted in the thinking around pay and holding back wages is the subtle belief that an employment opportunity is an opportunity to earn wages (considered a social good) irrespective of one’s input. The opportunity to work in a government establishment is perceived as a chance to earn from the bountiful government resources without much input. Individuals and communities who view things in this light may condone and inadvertently propagate absenteeism. If sanctions meted against a worker for absenteeism leads to pay being withheld, managers who enforced sanctions may face a backlash.

Importantly, the narratives also imply that absenteeism can be tolerated – or not, depending on the reasons. Generally, absenteeism arising from inadequate infrastructure, low wages and demands to fulfil other duties was considered tolerable. Health workers also exploited their knowledge about staff levels to reorganise shifts, leaving health centres understaffed. Although not knowing the staff levels is a norm in many countries, the findings allude to how asymmetry of information allows deviant health workers to be absent by adjusting duty rosters so that only one worker is in work at a given time while the rest of the workers are absent. Health workers were less tolerant of absenteeism which occurred when absent staff were backed by powerful persons, typically politicians, and so had no means of sanctioning them. Thus, socio-political systems have influenced understandings of what type of absenteeism, or whose absenteeism, is sanctionable. We highlighted the local power dynamics, understandings, and transactional relationships that interplay to institute, entrench, and normalise absenteeism. This is the most interesting dynamic as it suggests that interventions aiming to reduce absenteeism must recognise local meanings and realities. Generally, we found that absenteeism persists when health workers are confident that they are safe from sanctions.

As noted, both service users and health workers suggested (implicitly and explicitly) that absenteeism can be condoned when health workers are absent because they have to play sociocultural roles (e.g. child care), or that they are obstructed by infrastructural problems (e.g. transportation challenges), or they are trying to make up for their low/irregular wages. Other studies have found similar dynamics where gender roles caused female health workers to be absent, and their absence tolerated in work [33]. Health workers advance an internal understanding about these local realities and roles and adjust their schedules and activities to suit them. The different challenges faced by health workers gave rise to behaviours that allow them to meet their personal needs and somehow manage to keep the health centres running albeit sub-optimally. Service users also understand these limitations and are disposed to bear with health workers whose absence genuinely reflects these concerns. Health system factors such as low/delayed wages have been found to contribute to public health worker absenteeism, especially in low resource settings [34]. Thus, many dysfunctional acts adopted as a solution to dysfunctional health systems are normalised [5,29]. This tendency for frontline actors to take pragmatic approaches has been explained in the Corruption Functionality Framework [35], which describe corrupt behaviours to arise and be entrenched where they provide a ‘functional solution’ to existing problems. Even general corruption outside the health system impacts the health system [29]. We accept that corruption, and absenteeism as one of its forms, is not an individual but a systemic problem. But we argue that it is also rooted in history, established relationships, and social norms. It is important to understand the local narratives, making of meanings, and how people understand and rationalise absenteeism and its perceived impact.

Although health workers and community groups felt powerless about what to do with politically backed health workers, we found that some facility managers stood up to those absent without authorisation and challenged their behaviour and their political backers. People who do this, especially considering the context, are courageous, though sometimes they count on their political affiliates to protect them too. We also found reports that backlashes are real and sometimes workers who stand up to absenteeism face real threats to their lives and their jobs. Hence policies needed to address absenteeism must ensure that policy enforcers are guaranteed protection. We also found that some health managers profiteered off absentee health workers by demanding bribes and covering-up for them. We did not find a reason why some health managers acted differently, but we suspect that socio-psychological differences could be a reason.

Our findings have implications for interventions targeting absenteeism policies in Nigeria and other LMICs. Interventions that are not sensitive to the contextual realities and nuances will likely fail. We note that health worker absenteeism can be both tolerable and intolerable depending on the factors driving absenteeism but, nonetheless, both types negatively impact the health system. Politically backed absenteeism seems to be the most harmful form, rendering managers powerless to enforce rules. We argued that understanding local actors’ experiences with absenteeism is an important step to identify systemic inefficiencies as well as opportunities to engage with political figures and structures that may be motivated to address health worker absenteeism. This can lead to pragmatic strategies and mechanisms that can operate in the Nigerian context and fit with the socio-political norms and behaviours. Our findings suggest that health workers who follow the rules, and powerful community stakeholders within and outside the health facility committees could have important roles to play.

We must note certain limitation of the study. It would have been useful to juxtapose the findings with actual scale of absenteeism and to follow up health workers who are chronically absent. While data are lacking, self-reported experiences of absenteeism have been found to be a good measure among health workers [36]. Our findings are specific to the context of Enugu state and may not reflect the situation elsewhere in Nigeria, due to differences in local government regulations, as well as sub-national policies such as degree of enforcement of sanctions and leadership styles. However, we believe based on the literature, that the themes we identified may resonate with those in other settings and thus inform further research.

## CONCLUSION

In conclusion, we examined the narratives of health workers, managers, and service users constructed to explain absenteeism of health workers in Nigeria. Our key message is that although absenteeism is recognised as a vice, social realities influence which type of absenteeism, and which perpetrator is condoned. Absenteeism backed by politics and bribery are loathed, and attempts were made by actors to address it. Politically protected health workers who are absent are often not visible to community members, but their absence also impacts the quality of care in health centres as coworkers suffer stress and burnout. Interventions targeting absenteeism should think of two broad categories of absenteeism drivers- health system problems and political interference.
